# Synthesis and Evaluation of a Silver Nanoparticle/Polyurethane Composite That Exhibits Antiviral Activity against SARS-CoV-2

**DOI:** 10.3390/polym14194172

**Published:** 2022-10-04

**Authors:** Wing T. Lam, Tahkur S. Babra, Julian H. D. Smith, Mark C. Bagley, John Spencer, Edward Wright, Barnaby W. Greenland

**Affiliations:** 1 Department of Chemistry, School of Life Sciences, University of Sussex, Falmer, Brighton BN1 9QJ, UK; 2 AWE Plc, Aldermaston, Reading RG7 4PR, UK; 3 HungryTech Ltd. C/O Sussex Drug Discovery Centre, School of Life Sciences, University of Sussex, Falmer, Brighton BN1 9QG, UK; 4 Sussex Drug Discovery Centre, School of Life Sciences, University of Sussex, Falmer, Brighton BN1 9QG, UK; 5 Viral Pseudotype Unit, School of Life Sciences, University of Sussex, Falmer, Brighton BN1 9QG, UK

**Keywords:** anti-viral coatings, silver nanoparticles, nanocomposite, polyurethane, COVID-19, SARS-CoV-2

## Abstract

In this proof-of-concept study, we aim to produce a polyurethane (PU)-based composite that can reduce the amount of viable SARS-CoV-2 virus in contact with the surface of the polymeric film without further interventions such as manual cleaning. Current protocols for maintaining the hygiene of commonly used touchpoints (door handles, light switches, shop counters) typically rely on repeated washing with antimicrobial products. Since the start of the SARS-CoV-2 pandemic, frequent and costly surface sanitization by workers has become standard procedure in many public areas. Therefore, materials that can be retrofitted to touchpoints, yet inhibit pathogen growth for extended time periods are an important target. Herein, we design and synthesise the PU using a one-pot synthetic procedure on a multigram scale from commercial starting materials. The PU forms a robust composite thin film when loaded with 10 wt% silver nanoparticles (AgNPs). The addition of AgNPs increases the ultimate tensile strength, modules of toughness and modulus of elasticity at the cost of a reduced elongation at break when compared to the pristine PU. Comparative biological testing was carried out by the addition of pseudotyped virus (PV) bearing the SARS-CoV-2 beta (B.1.351) VOC spike protein onto the film surfaces of either the pristine PU or the PU nanocomposite. After 24 h without further human intervention the nanocomposite reduced the amount of viable virus by 67% (*p* = 0.0012) compared to the pristine PU treated under the same conditions. The significance of this reduction in viable virus load caused by our nanocomposite is that PUs form the basis of many commercial paints and coatings. Therefore, we envisage that this work will provide the basis for further progress towards producing a retrofittable surface that can be applied to a wide variety of common touchpoints.

## 1. Introduction

Severe acute respiratory syndrome coronavirus-2 (SARS-CoV-2) is the aetiological agent of coronavirus disease 2019 (COVID-19). It is the first pandemic of the 21st century caused by a coronavirus (CoV) [1]. It has dramatically affected the lives of people across the world since report of the first cases in late 2019, and subsequent identification in early 2020 [2]. At the time of writing, COVID-19 has been estimated to have infected over half a billion people, and claimed over 6 million lives, numbers that are likely be an underestimation and will to continue to grow [3]. Although many of the most serious effects of the virus have been mitigated in some countries by high uptake of vaccines and improved treatment regimens, access to these measures is not spread equally across the globe.

The route of transmission of a virus is one of the key features in determining the speed at which it can spread through a population. Transmission between humans can be through multiple mechanisms, and for SARS-CoV-2 the major route is thought to be via aerosolised droplets, produced whilst coughing, sneezing or talking, which can each contain millions of virus particles [4]. However, it is also possible that any shed virus that has settled on a surface can persist for many hours or even days [5,6]. While this is not thought to be the main mechanism by which this virus is spread, contact with these contaminated surfaces (fomites) can also facilitate transmission [7]. Fomite transmission may be the major means of viral spread during future pandemics. Therefore, designing surfaces which reduce viability of viruses they come into contact with are a pressing scientific concern [8], especially if this means the surface does not have to be repeatedly sanitised by, for example, washing with antimicrobial agents. These self-sanitizing surfaces [9] would be especially important during early stages of a pandemic when such non-pharmaceutical interventions are the only means to control the spread of infection.

It is known that the nature of the surface, for example its material properties such as porosity [10,11] as well as the specific chemistry of the surface (e.g., presence of ions [12,13]), can heavily influence the viability of viruses on surfaces. Many heavy metals have been shown to exhibit antiviral properties [14]. A range of metal oxides, for example zinc [15,16], magnesium [17], copper [18] and titanium [19] often in the form of nanoparticles (NPs), have also been demonstrated to reduce the persistence time for a range of microbes and viruses on surfaces. With respect to COVID-19, early work in the area showed that solid copper surfaces (for example door handles) dramatically reduce the persistence time of viable viruses over an 8 h period compared to typical alternatives such as stainless steel or ‘plastic’ [5]. Amongst metal NPs, silver NPs (AgNPs) have received the most study concerning antiviral activity [20,21,22,23,24,25]. Specifically with respect to SARS-CoV-2 AgNPs, they been demonstrated to reduce the viral load when coated on the surface of fabrics, e.g., surgical masks [26].

NPs (fillers) can also be added to polymers (continuous phase) to form nanocomposites which have been investigated for a range of applications, for example to produce healable materials [27,28] or debond-on-demand adhesives that respond to EM radiation [29]. Although the physical properties (e.g., tensile modulus, ultimate tensile strength) of the polymer changes on addition of the NPs, frequently the properties of the NPs (e.g., antimicrobial [30]) are retained in nanocomposite polymer. It can be envisaged that a nanoparticle containing coating material that can cover preinstalled touchpoints to give antiviral properties would offer significant cost advantages over preplacing these touchpoints with solid metal analogues that have been shown to reduce viral load (e.g., covering steel door handles rather than replacing them with copper door handles). Therefore, the key design criterion of a retrofittable nanocomposite surface coating is that the AgNPs are delivered in a form that allows them to stick to most surfaces (for example touchpoints: door handles, handrails and countertops) whilst retaining their antiviral activity. This process would make the surface instantly self-sanitizing without the cost of changing the entire touchpoint or repeated sanitization throughout the day.

The antiviral mechanism for AgNPs depends on the virus or microbe in question and is reported [31] to be as a consequence of the Ag+ ions interfering with either the electron transport system or pathogen nucleic acid [32]. Regardless of mechanism, the antimicrobial activity of AgNPs without the ability for pathogens to gain significant resistance is well documented. When combined with the very low toxicity profile of AgNPs towards mammals including humans [33,34], these features make AgNPs attractive materials for self-sanitizing surfaces [35].

As a starting point for this project, a polyurethane (PU) [36] was selected as the continuous phase of the nanocomposite. The solubility, inexpensive nature of the starting materials and the ability for PUs in general to form tough films has seen them become one of the most important materials in the paints and coatings industry [37]. PUs are synthesised from step growth polymerisation of oligomeric diol components with diisocyanates which results in an alternating linear co-polymeric structure [38]. The physical properties of PUs can be tuned by altering the structure of the diol, diisocyanate or by the addition an end group that can affect the morphology of the bulk material. For example, work from our laboratory [39] and the Hayes [40,41] group amongst others [42] has studied the structure/property relationships in supramolecular PUs. Structurally simple, supramolecular materials [43,44,45,46] can typically be produced from a three-component feedstock: (i) a diisocyanate; (ii) oligomeric diol and (iii) a hydrogen bonding chain end. Varying the hydrogen bonding propensity of the chain end results in a significant change in the physical properties of the resulting bulk material [40,47].

Our objectives for this study focus on the design, synthesis and evaluation of a potential coating material that exhibits the properties required to form the basis of a retrofittable self-sanitising surface. Ultimately, our resulting PU forms self-supporting films on solution casting and composite PU/AgNPs films are shown to reduce the viable titre of SARS-CoV-2 that remains on the surface for 24 h by *c.* 70% compared to analogue PU surfaces that lack AgNPs [48].

## 2. Materials and Methods

### 2.1. Materials

Unless stated otherwise, chemicals and solvents were purchased from Merck (Gillingham, UK). Anhydrous tetrahydrofuran (THF), 4,4′-methylene bis(phenyl isocyanate) (98%), 4-(2-aminoethyl)morpholine (99%) were used as received. AgNPs (Merck, Gillingham, UK) were less than 100 nm in diameter. Krasol^TM^ HLBH-P2000 was supplied by Cray Valley, Saint- Avold, France.

### 2.2. Characterisation

^1^H (600 MHz) and ^13^C NMR (150 MHz) spectra were recorded on a Varian NMR 600 MHz spectrometer at room temperature, using the residual protic solvent signal in the deuterated solvent for calibration (chloroform-*d* at δ 7.26 ppm). Chemical shifts are reported in ppm. Spin multiplicities are reported as a singlet (s), doublet (d), triplet (t) or multiplet (m) with coupling constants (*J*) given in Hz, where applicable. Tensile stress–strain experiments were carried out using an AML X5-500 single column universal tester, equipped with a 5 kN load cell and wedge grips. Specimens with dimensions of 40 mm × 5.0 mm × *c.* 0.3 mm were cut from the films for testing. The samples were analysed at a strain rate of 100 mm min^−1^. The instrument was calibrated according to the requirements of ISO 7500-1. Size exclusion chromatography (SEC) was conducted using a Polymer Laboratories PL-GPC 220 high temperature chromatograph equipped with PL Mixed Gel columns at 40 °C eluting with analytical grade chloroform. Samples were dissolved in the same solvent (at 5 mg mL^−1^ concentration) and filtered through a syringe filter (0.2 µm) prior to injection. Molecular weight data were processed using standard Agilent GPC/SEC software and are reported in comparison to ‘PL Easy-Cal’ polystyrene standards.

### 2.3. Synthesis of **PU1**

Krasol^TM^ HLBH-P2000 (4.5 g, 2.25 mmol) was heated under vacuum at 120 °C for 1 h. Methylene diphenyl diisocyanate (1.13 g, 4.5 mmol) was added to the stirred polymer in one portion under argon at 80 °C for 3 h, followed by a solution of (2-aminoethyl)-morpholine (0.64 g, 4.95 mmol) in dry THF (25 mL). After 6 h at 50 °C most of the THF was removed in vacuo and MeOH (50 mL) was added to the resulting viscous polymer solution, resulting in the precipitation of the polymer as a sticky solid on the edge of the flask. The solvents were decanted and the polymer dried to give **PU1** as a pliable colourless solid 4.57 g (73%). ^1^H NMR (600 MHz, CDCl_3_) δ 0.82–1.65 (276H, m), 2.44 (8H, m), 2.49 (4H, t, *J* = 6.0), 3.33 (4H, q, *J* = 6.0), 3.60–3.64 (8H, m), 4.12–4.17 (6H, m), 5.35 (br), 6.57 (br), 7.10–7.20 (11H, m); ^13^C NMR (151 MHz, CDCl_3_) δ 137.1, 136.4, 130.0, 129.9, 129.7, 122.0, 121.8, 77.2, 67.2, 53.7, 41.0, 39.2, 38.7, 38.2, 36.4, 33.8, 33.6, 31.0, 30.6, 30.1, 27.1, 26.9, 26.2, 11.2, 11.0. SEC (Chloroform, 40 °C), *Mn* = 8700 g mol^−1^ and *Mw* = 15,400 g mol^−1^.

### 2.4. Film Casting and Composite Formation

Uniform thickness films of **PU1** were prepared by dissolving **PU1** (1.6 g) in of THF (8 mL) at room temperature. This was poured into a Petri dish (diameter 140 mm) and left at room temperature for 1 h. Subsequently, the tacky film was place in a vacuum oven at 50 °C and 800 mbar for 18 h. Upon cooling to ambient temperature, the film (0.32 mm thick) could be removed from the Petri dish. The nanocomposite film, **PU1/AgNP**, (0.34 mm thick) was formed by following the same casting procedure using 3.0 g of polymer dissolved in 15 mL of THF to which 0.30 g of AgNPs were added. Film thicknesses were determined using Vernier callipers.

### 2.5. Biological Testing

Prior to testing for antiviral activity, membranes were sterilised by treating with 70% ethanol and exposure to UV light for 1 h. Luciferase encoding lentiviral pseudotypes were prepared as previously described (PMID: 34859134). These were titrated onto HEK293T cells stably expressing ACE2 and TMPRSS2 (NIBSC Repository, UK. With thanks to Dr. Leila Mekkaoui and Martin Pule, Autolus Ltd. London, UK) to determine viral titres. Within a single well of a 6-well plate, 50 µL of the cell supernatant containing pseudotyped virus (PV) bearing the beta (B.1.351) spike protein was then spotted onto the required polymer or composite film. This was incubated for 24 h at room temperature. Following this, 1.5 × 10^5^ HEK293T+ACE2+TMPRSS2 (human embryonic kidney cells + angiotensin-converting enzyme 2 + transmembrane protease serine 2) were added to the PV and membrane, and the plate was incubated at 37 °C for 1 h (5% CO_2_). The membrane was then removed and the plate incubated at 37 °C (5% CO_2_) for a further 48 h. Following this a 50:50 mix of Bright-Glo (Promega, UK) and serum-free media was added to each well and the reporter activity assessed using a Glomax Explorer (Promega, Southampton, UK).

## 3. Results and Discussion

### 3.1. Polymer Design and Synthesis

**Scheme 1:**
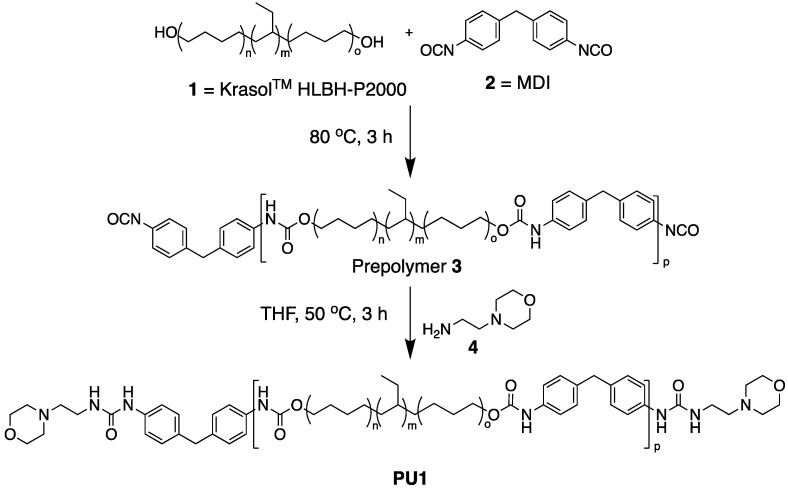
Synthesis of **PU1**, n + m + o ≈ 35, p ≈ 3.

Morpholine terminated PU (**PU1**, Scheme 1) was selected as a suitable starting point for this work. It is known that chemically related structures are adhesive and can be cast into self-supporting films [44], and also can form composite materials containing NPs [27], all features that are required for this self-sanitising, retrofittable surface.

**PU1** contains Krasol^TM^ HLBH-P2000 (diol component), 4,4′-methylene diphenyl diisocyanate (MDI) (**2**) and 4-(2-aminoethyl)morpholine (**4**) (the end group). Krasol^TM^ HLBH-P2000 is a random co-polymeric diol produced by the reduction of a polybutadiene that contained linear and branched diene residues. To maintain the potential ease of future scale up of this synthesis, starting materials and solvents were used as received rather than adding costly and time-consuming purification steps prior to commencing the synthesis.

The synthesis of **PU1** was carried out using an established one-pot, two step procedure. Firstly, isocyanate prepolymer (**3**) was synthesized by addition of equimolar quantities of MDI (**2**) to neat Krasol^TM^ HLBH-P2000 **1**. After 3 h at 80 °C, the reaction was cooled to 50 °C and a solution of 4-(2-aminoethyl)morpholine (**4**) in THF was added to endcap the prepolymer [47]. The polymer was isolated by precipitation from THF into methanol (73% yield).

### 3.2. Polymer Characterisation

**Figure 1:**
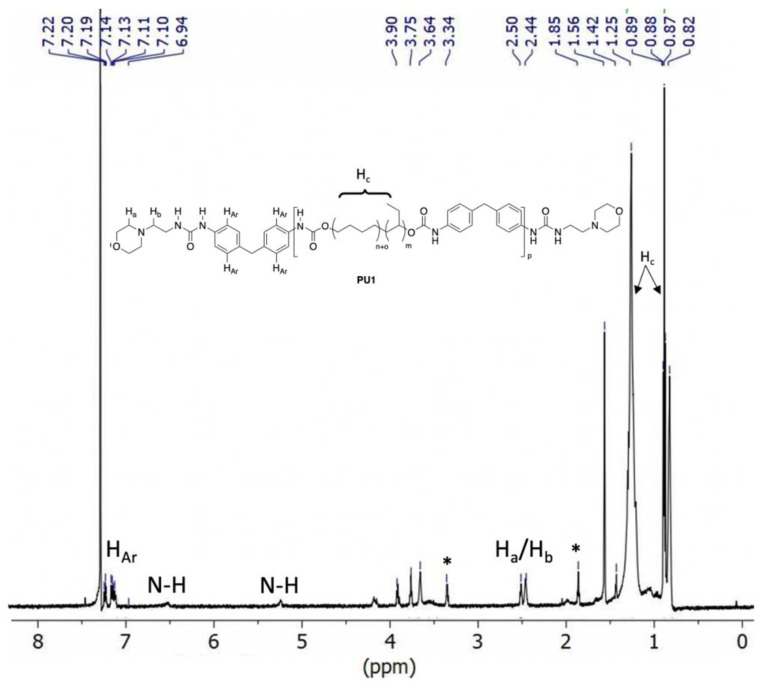
^1^H NMR spectrum (600 MHz, CDCl_3_) of **PU1** with selected assignments. * = signals from residual THF in the polymer after drying.

Analysis of the ^1^H NMR spectrum of the polymer (**PU1**, Figure 1) shows resolved signals for each of the structural features expected in the target product. The aliphatic residues of the Krasol^TM^ (H_c._ in Figure 1) are readily apparent as intense signals at δ 0.8 and 1.3 ppm. Signals indicative of the aromatic protons from the MDI starting material appear at δ 7.2 ppm (H_Ar_), and signals for the end groups are well resolved, for example H_a_ and H_b_ can be seen at approximately δ 2.5 ppm. Successful formation of the urethane and urea functionalities is supported by the broad signals at δ 5.2 and 6.5 ppm which are indicative of N-H protons.

Analysis of **PU1** by size exclusion chromatography (SEC) in chloroform gave *Mn* = 8700 g/mol and *Mw* = 15,400 g/mol compared to narrow dispersity polystyrene standards. The resulting dispersity value (Ð) of 1.8, is typical of polymers produced by step growth polymerisations. The relatively low molecular weight of the product may be as a consequence of the nominal molecular weight of the Krasol^TM^ diol (*c.* 2000 g/mol) monomer which precludes accurate stoichiometry matching of the starting materials during synthesis of the prepolymer. In addition, we add end group to facilitate hydrogen bonding in the product which also serves to stop the polymerization proceeding.

**Figure 2:**
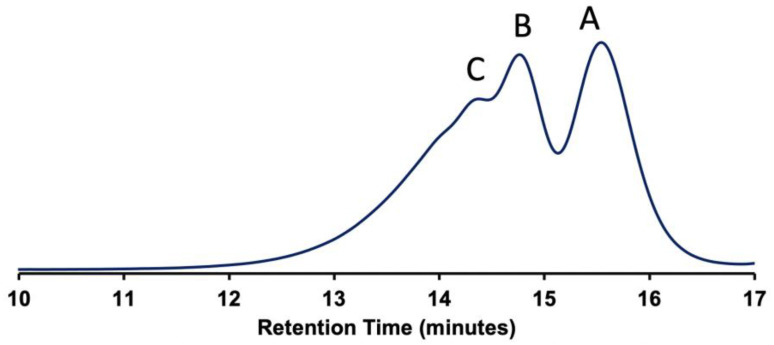
SEC eluogram (5 mg/mL, chloroform, at 40 °C) showing resolution of a series of species containing 1, 2 and 3 Krasol^TM^ residues (peaks (A), (B) and (C)).

Inspection of the SEC eluogram showed a multimodal molecular weight distribution profile, with three well resolved maxima at retention times between 14 and 16 min, (Figure 2). These signals are indicative of structures containing 1, 2 and 3 Krasol^TM^ diol residues (i.e., *p* = 1, 2 and 3 for **PU1** in Scheme 1). The ability of these three oligomers to be resolved by SEC is as a consequence of their significant difference in molecular weight (*c*. 2300 g/mol between each member of the series). Similar resolution by SEC of low *p* value PUs has been observed previously for structurally related PUs [48].

## 4. Composite Formation and Film Casting

### 4.1. Film Casting and IR Spectroscopic Analysis

**Figure 3:**
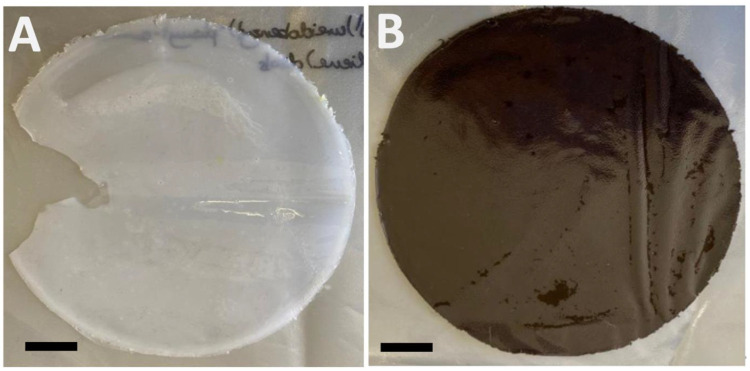
Photographs of the solution cast films of: (**A**) **PU1** and (**B**) **PU1/AgNP** composite. Scale bars = 1 cm.

Uniform films (*c*. 0.3 mm thick) of **PU1** and **PU1** containing 10 wt% AgNPs (**PU1**/**AgNP**) were readily cast from THF by slow evaporation of the solvent at 50 °C at 800 mbar for 18 h (Figure 3). Efforts to speed up the casting process by exposing the materials to higher temperatures or vacuums resulted in films that contained multiple air pockets, and that were not suitable for further analysis or testing.

The FTIR spectrum for **PU1** did not contain a signal at 2255 cm^−1^, which would be indicative of unreacted isocyanate, but contained a broad absorbance band characteristic for NH bonds centred at 3309 cm^−1^ (See Supplementary Materials). The spectrum also showed a signal for the carbonyl groups at 1678 cm^−1^, which is consistent with urethane formation. The signal for these groups were significantly red shifted in the spectrum of **PU1**/**AgNP** (3267 cm^−1^ and 1645 cm^−1^ see Supplementary Materials). This shows that the AgNPs were interacting with the polymer through both the NH and C=O functional groups [49].

### 4.2. Strength Testing

**Figure 4:**
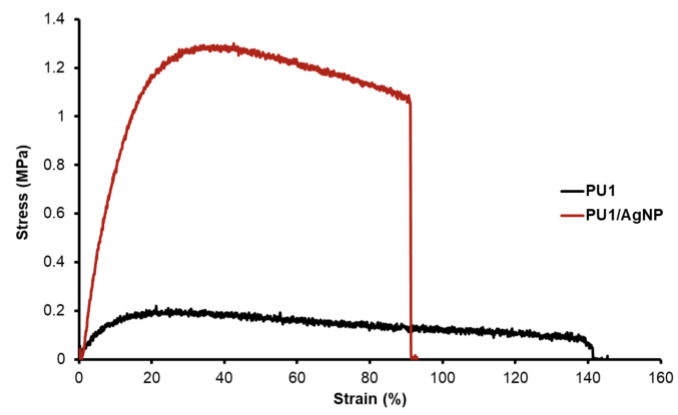
Representative stress–strain plots for **PU1** (black) and **PU1/AgNP** (red).

Mechanical testing was conducted on **PU1** and the **PU1/AgNP** composite. In each case 5 samples with dimensions 40 mm × 5.0 mm × *c.* 0.3 mm were cut from the solution cast polymer films (Figure 3), then analysed by stress–strain analysis at a strain rate of 100 mm/min (for calculation equations and methods see Supplementary Materials). Representative stress–strain data for the two materials are shown in Figure 4. Table 1 shows mean values calculated from stress–strain data for 5 independent tests of each material.

As can be seen visually from the stress–strain plot and is readily apparent from the mean values shown in Table 1, addition of the AgNPs to PU1 had a dramatic effect on the mechanical properties of the material (for calculation methods and equations see the Supplementary Materials). Both materials showed ductile properties with **PU1** exhibiting the higher extension at break (**PU1** = 243% and **PU1/AgNP** = 53%). The significant plastic deformation observed in the stress strain curves may be as a consequence of entrapped residual THF which would serve to plasticise the sample during strain testing.

The composite exhibited a significantly higher modulus of toughness compared to **PU1** (0.54 vs. 0.22 MPa), tensile modulus (2.4 vs. 10.9 MPa), and UTS (0.18 vs. 1.20 MPa). As expected, therefore, the addition of a nanoparticulate filler increases the resistance to deformation at the cost of reduction in elongation at break. Elongation at break is not a significant parameter in a coatings technology as it would be expected that the base material which was coated (door handles, other touch points) would prevent elongation.

### 4.3. Biological Data

**Figure 5:**
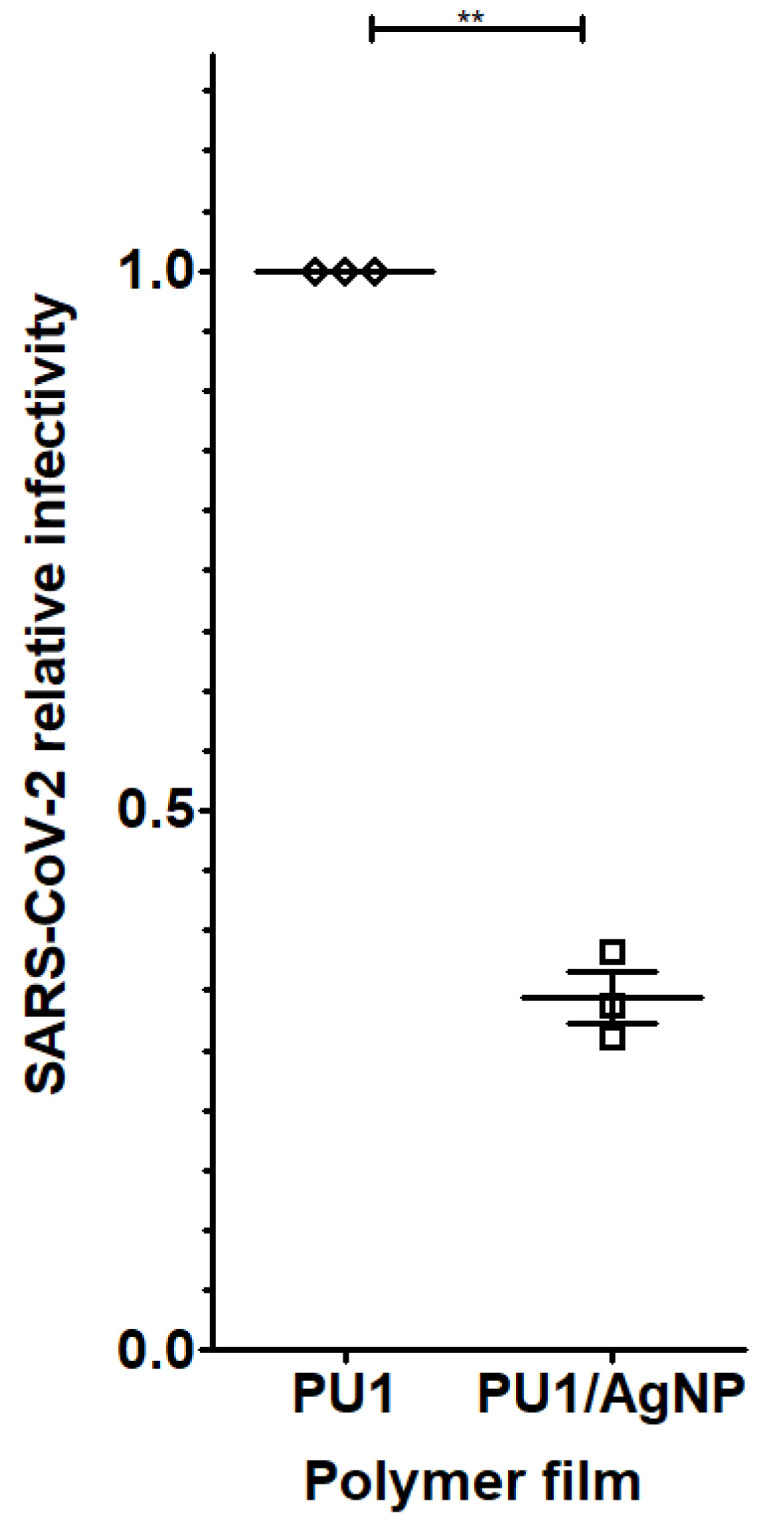
Inhibition of SARS-CoV-2 infectivity by **PU1/AgNP**. The relative infectivity of SARS-CoV-2 that had been incubated on **PU1** or **PU1/AgNP** was compared. Significance was determined using a *t*-test (**—*p* = 0.0012).

In order to assess the antiviral activity of the polymer films they were used in viral infectivity assays. This biological testing was carried out in a six well plate with the wells containing strips of **PU1** or **PU1/AgNP** composite (*c*. 1 cm × 1 cm). 50 µL of the cell supernatant containing pseudotyped virus (PV) bearing the SARS-CoV-2 beta (B.1.351) VOC spike protein was then spotted onto the surface of the films, and then incubated for 24 h before the target cells were added. At the conclusion of the incubation period a 50:50 mix of Bright-Glo and media was added to each well and the reporter activity assessed using a Glomax Explorer. This complete procedure was repeated three times, with four luciferase readings taken for each condition for each repeat. The ability of the **PU1/AgNP** composite film to inhibit SARS-CoV-2 was then calculated by comparing the level of virus infection achieved to that observed with the **PU1** film. The results, Figure 5, show that the **PU1/AgNP** composite film, reduces the amount of viable virus 67% (*p* = 0.0012).

## 5. Conclusions

In conclusion, we have synthesised a polyurethane (**PU1**) in a high yielding one pot, two step procedure that can readily incorporate AgNPs to form a composite material. The composite material (**PU1/AgNP**) exhibits tensile modulus, ultimate tensile strength, and modulus of toughness values of 1.2 MPa, 10.9 MPa and 0.52 MJm^−3^, respectively, each of which is higher than those measured for **PU1** which were 0.18 MPa, 2.37 MPa and 0.22 MJm^−3^. In contrast, the elongation at break measure for **PU1/AgNP** was significantly less than that observed for **PU1** (53 vs. 243%). The ability of films cast of **PU1** and **PU1/AgNP** to inhibit SARS-CoV-2 infectivity was assessed by biological infection assay. **PU1/AgNP** films were found to reduce the amount of viable virus by 67% (*p* = 0.0012) compared to **PU1**.

PUs are the basis for a range of commercially viable paints and coatings and, therefore, this work shows the potential for PUs to form the basis of retrofittable surface coatings that reduce fomite transmission of SARS-CoV-2.

Work is underway to investigate how the dispersion of NPs effects the anti-viral properties of the film. We also propose that the chemical structure of the PU (hydrophobicity, hard/soft domain size, etc.) will have a significant impact on SARS-CoV-2 inhibition. In addition, the chemical structure of the PU will also clearly impact the overall toughness of the film, which is an important factor in producing a viable coating, and these features are the focus of continuing studies in the lab.

## Figures and Tables

**Table 1:** Mean values from stress–strain analysis data for **PU1** and **PU1/AgNP** (*n* = 5). Standard deviation (s.d.) is calculated from the data from 5 independent measurements (*n* = 5).

Material	Ultimate Tensile Strength, MPa (s.d.)	Tensile Modulus, MPa (s.d.)	Modulus of Toughness, MJ m^−3^ (s.d.)	Elongation at Break % (s.d)
**PU1**	0.18 (0.02)	2.37 (0.56)	0.22 (0.04)	243 (106)
**PU1/AgNP**	1.20 (0.26)	10.87 (3.81)	0.52 (0.32)	53 (27)

## Data Availability

Not applicable.

## Supplementary Materials

The following supporting information can be downloaded at: https://www.mdpi.com/article/10.3390/polym14194172/s1, Figure S1: FTIR spectra of PU1 and PU1/AgNP; Figure S2: ^13^C NMR spectrum of **PU1** (150 MHz, CDCl_3_); Stress Strain Calculation Methods and Equations.
