# Supplementary material for: Synthesis and Evaluation of a Silver Nanoparticle/Polyurethane Composite That Exhibits Antiviral Activity against SARS-CoV-2
Source: Polymers (Basel). 2022 Oct 4;14(19):4172. doi: 10.3390/polym14194172 (PMC9571720; doi:10.3390/polym14194172)

Synthesis and evaluation of a silver nanoparticle/polyurethane composite that exhibits antiviral activity against SARS-CoV-2.

FTIR spectra of PU1 and PU1/AgNP **Page S1**

Stress Strain Calculation Methods and Equations **Page S2**

^{13}C NMR spectrum of PU1 **Page S3**

Figure S1. FTIR spectra of **PU1** (green – top spectrum) and **PU1/AgNPs** (Blue – lower sprectrum).

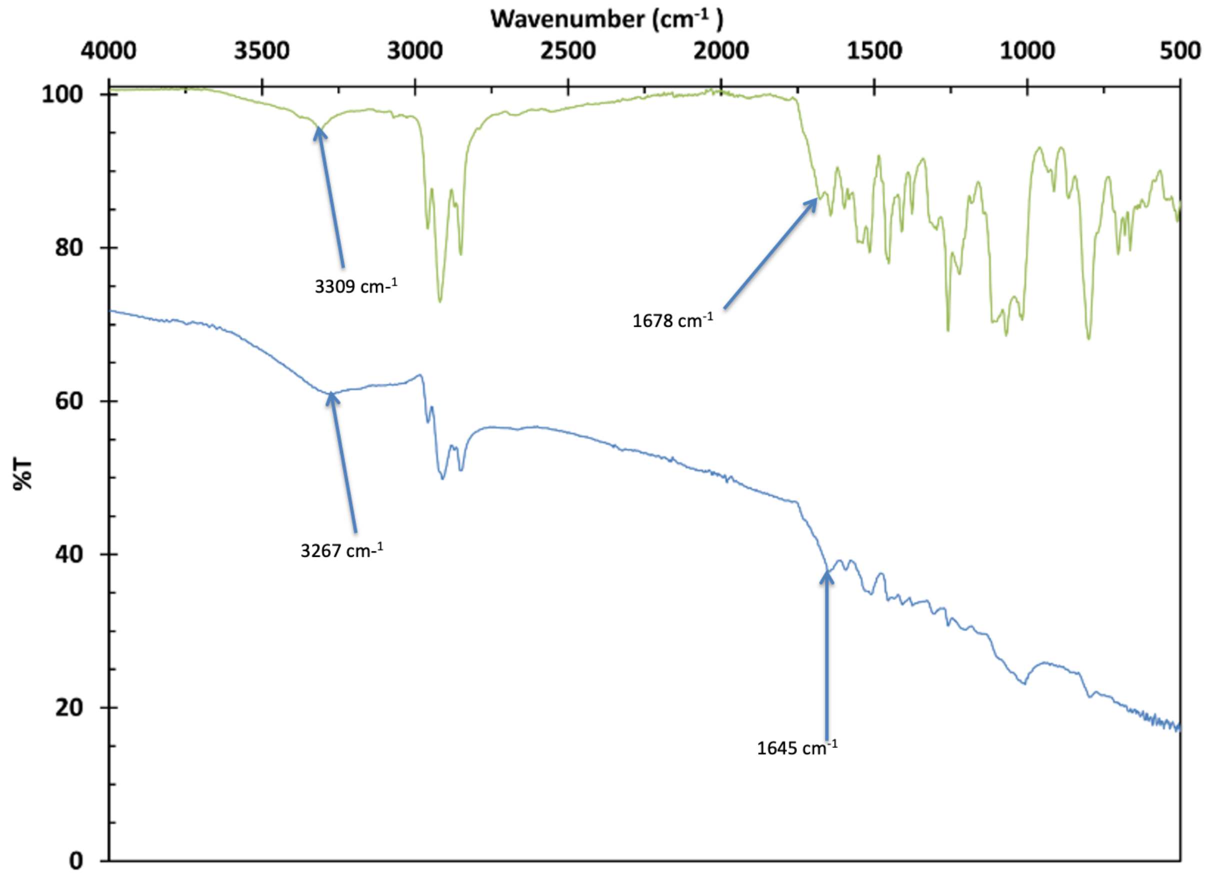

Stress Strain Calculation Methods and Equations:

The maximum force at break (N) was taken from the recorded data without any further calculation

The Ultimate Tensile Strength (UTS) (MPa) was calculated using the following equation:

$$UTS\ (MPa) = \frac{Maximum\ Force\ (N)}{Cross\ sectional\ area\ of\ the\ pristine\ sample\ (mm^2)}$$

Young's Modulus is calculated from the gradient of the force x extension graph at initial force using the equation.

$$Young's\ Modulus\ (MPa) = \frac{Change\ in\ Force\ (N)}{Change\ in\ extension\ (mm^2)}$$

The Elongation at break (EaB) is calculated by taking the change in extension of the sample at break and dividing by the initial length of the material, and reported as a percentage.

$$EaB\ (\%) = \frac{Elongation\ at\ break - Length\ of\ Original\ Sample\ (mm)}{Length\ of\ Original\ Sample\ (mm)} \times 100$$

The Modulus of Toughness is the area under the stress strain graph, and can be calculated by using the trapezium rule; where by the area of the graph is split into trapeziums at each consecutive point, and the sum of the area of each trapezium is totalled to give the Modulus of Toughness.

$$Modulus\ of\ Toughness\ = \sum(0.5\ \times (Stress\ A + Stress\ B)\ \times (Strain\ B - Strain\ A))$$

Figure S2. ^{13}C NMR spectrum of PU1 (150 MHz, CDCl3).

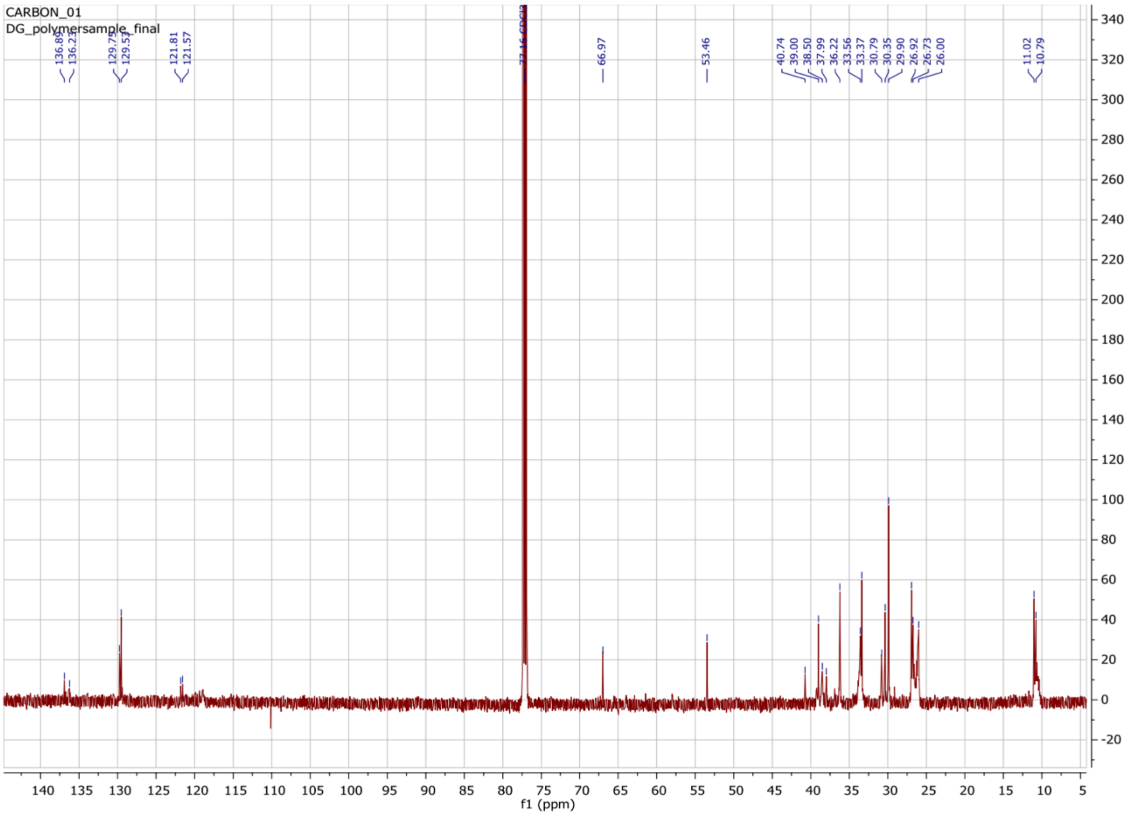

Supplement: Supplementary file 1 [file polymers-14-04172-s001.zip › polymers-1909651-supplementary.pdf]
